# Natamycin versus natamycin combined with voriconazole in the treatment of fungal keratitis

**DOI:** 10.12669/pjms.39.3.6908

**Published:** 2023

**Authors:** Jingjing Cai, Chengwen Yang, Qiuhong Wei, Huifang Lian, Lin An, Rong Zhang

**Affiliations:** 1 Jingjing Cai, Department of Ophthalmology, Baoding No.1, Central Hospital, Baoding 071000, Hebei, China; 2 Chengwen Yang, Department of Ophthalmology, Baoding No.1, Central Hospital, Baoding 071000, Hebei, China; 3 Qiuhong Wei, Department of Ophthalmology, Baoding No.1, Central Hospital, Baoding 071000, Hebei, China; 4 Huifang Lian, Department of Ophthalmology, Baoding No.1, Central Hospital, Baoding 071000, Hebei, China; 5 Lin An, Department of Ophthalmology, Baoding No.1, Central Hospital, Baoding 071000, Hebei, China; 6 Rong Zhang, Department of Ophthalmology, Baoding No.1, Central Hospital, Baoding 071000, Hebei, China

**Keywords:** Natamycin, Voriconazole, Fungal keratitis, Corneal ulcer

## Abstract

**Objective:**

To observe the therapeutic effect of natamycin combined with voriconazole in the treatment of fungal keratitis (FK).

**Methods:**

This is a retrospective study. The subjects of this study were 64 patients with FK who were admitted to Baoding No.1 Central Hospital from February 2019 to July 2022. The enrolled patients were divided into control group (*n*= 32) and study group (*n*= 32) by the random number table method. The control group was treated with natamycin alone, and the study group was treated with natamycin combined with voriconazole. The total efficacy, time of disappearance of ocular symptoms, visual acuity level, keratitis severity score, corneal ulcer area, tear fungus index and incidence of adverse reactions were compared between the two groups.

**Results:**

The total efficacy,of the study group was significantly higher than that of the control group. The disappearance time of corneal ulcer, photophobia, foreign body sensation and hypopyon in the study group was shorter than those in the control group. Keratitis severity score and (1,3)-β-D-glucan level in the study group were lower than those in the control group. The corneal ulcer area was narrowed in the study group than that of the control group, and the visual acuity level in the former group was higher than that of the latter group. Besides, there was no significant difference in the frequency of adverse reactions between the two groups.

**Conclusion:**

Natamycin combined with voriconazole is safe and effective in the treatment of FK.

## INTRODUCTION

Fungal keratitis (FK) is an ocular disease caused by pathogenic corneal infection after the fungal invasion (aspergillus, yeast, Candida, etc).^1^,^2^ It has no specific symptoms in the early stage, and it is difficult to differentiate from bacterial keratitis and viral keratitis clinically, leading to high rates of missed diagnosis and misdiagnosis. Consequently, some patients may have the risk of blindness owing to missing of the best opportunity for treatment, which seriously affects their daily life and work.^3^-^5^ At present, natamycin has been recognized to be the major therapeutic choice for FK. Natamycin has strong bactericidal and anti-fungal effects, which has been accepted as the first line drug for the treatment of FK.^6^ However, it has a poor effect on patients with deep corneal infection, which may be attributed to the limitation of monotherapy.

Accordingly, the approach of combined medication is proposed in the clinical setting. According to a previous report,^7^ Voriconazole is a triazole antifungal agent derived from fluconazole, which is characterized by broad-spectrum anti-fungal properties and good corneal permeability. It is highly sensitive to Aspergillus, Penicillium, Candida, Cryptococcus neoformans and other fungi. At present, Voriconazole has been recommended as the first choice for the treatment of fungal infection. With respect to the above, the present study was conducted to apply combine medication of natamycin and voriconazole for the treatment of FK to explore the clinical efficacy of the two drugs primarily.

## METHODS

This is a retrospective study. The subjects of the study were 64 patients (64 eyes) with FK who were admitted to Baoding No.1 Central Hospital from February 2019 to July 2022. The enrolled patients were divided into control group (*n*= 32, 32 eyes) and study group (*n*= 32, 32 eyes) by the random number table method. Among them, there were 17 males (17 eyes) and 15 females (15 eyes) in the control group, with a mean age of (36.59 ± 5.48) years old (ranging from 20 to 65 years old); The course of the disease ranged from seven days to five months, with an average of (2.23 ± 1.15) months. Besides, the study group had 16 males and 16 females, with a mean age of (35.77 ± 5.64) years old (ranging from 19 to 65 years old); The course of the disease ranged from five days to four months, with an average of (2.05 ± 1.24) months. The comparison of various data between groups indicated no statistical differences (*P*>0.05), suggesting comparability between groups. The study was approved by the Institutional Ethics Committee of Baoding NO.1 Central Hospital (No.: [2022]052; Date: November 03, 2022).

### Diagnostic criteria:^8^

(1) patients with positive results of the fungal culture of the corneal scraping; (2) patients with the corneal ulcer area >one mm^2^ by slit-lamp microscopic examination, with hypopyon and decreased visual acuity; (3) patients with positive results by potassium hydroxide wet mount; and (4) patients with hyphal infiltration by confocal microscopy of the cornea, and with effective antifungal treatment. Patients can be diagnosed with FK when meeting any of the above criteria.

### Inclusion criteria:

- Patients who met the above diagnostic criteria;
- Patients with monocular disease;
- Patients who were informed of the study and signed the consent form.

### Exclusion criteria:

- Patients with other bacterial infections;
- Patients at the risk of or with corneal perforation;
- Patients with other eye diseases, such as glaucoma, limbal stem cell abnormal eye disease, etc.;
- Patients who had been treated with other antibiotics before enrollment;
- Patients with history of allergy to the drugs used.;
- Patients with mental illness.

***The treatment method of the control group was as follows:*** patients in this group were provided with local administration of Natamycin eye drops (North China Pharmaceutical Co., Ltd.; State Medical Permit No.: H20083293) at the initial dose of one drop/time every one ~ two hour for three ~ four days, and then one drop for six ~ eight times for two weeks continuously. Patients in the study group were treated with voriconazole on the basis of the same treatment as the control group. The use method of self-made voriconazole eye drops was as follows: 100mg voriconazole for injection produced by Pfizer Limited was mixed and diluted with five ml special solvent and 5ml sterile water for injection to prepare voriconazole eye drops (volume of 10ml, concentration of 10g/L).

The preparation was processed following the principle of aseptic operation strictly. After successful preparation, the drug was stored in a refrigerator at 4 ± 2°C. A single bottle was generally used for two ~ four days(one drop/time, once every two hour) for two weeks. During the treatment, the time and interval of medication can be adjusted appropriately according to the improvement of the condition of the two groups of patients. Meanwhile, antibiotics, vitamins, atropine ophthalmic gel, non-steroidal anti-inflammatory agents, etc. can be used in combination according to the condition of illness.

### Observation indexes:

The clinical efficacy of patients in the two groups was compared and evaluated according to the clinical examination results after treatment. Patients were considered to be cured if there was no hypopyon or corneal ulcer in the eyes after treatment by slit-lamp biomicroscopes, with the visual acuity increased by ≥3 lines. The treatment was identified to be significantly effective if a small amount of hypopyon was found by slit-lamp biomicrography, the measured corneal ulcer area was reduced by >50%, and the visual acuity was increased by ≥two lines.

The treatment was effective when there existed relieved ocular signs by slit-lamp biomicrography, and the visual acuity was improved by one to two lines compared with the previous examination. The treatment was invalid when there was no change or even aggravation of the ocular sign and corneal ulcer, no increase or even decrease in the visual acuity.^9^,^10^ The sum of cure rate, significant effective rate and the effective rate was the total effective rate.

Further comparison was performed on the disappearance time of ocular signs (corneal ulcer, hypopyon, intra-ocular foreign body sensation, photophobia and tearing),^11^ visual acuity level,^12^ keratitis severity score, corneal ulcer area, tear fungus index, etc. The severity of keratitis was mainly evaluated by anterior chamber reaction, lesion size, lesion depth, etc. The scores of the three items ranged from zero to three points, and the score was directly proportional to the severity.^13^ Detection of tear fungus: the tears of patients were sucked using a sterile capillary pipette, which were sucked and tested before and after treatment for the detection of (1,3)-β-D-glucan level.^14^

The incidences of irritating pain, foreign body sensation, photophobia, endophthalmitis, conjunctival congestion and other adverse reactions were recorded and compared between the two groups during treatment.^15^

### Statistical analysis:

Data analysis of this study was achieved with SPSS 20.0 software. The measurement data was described by (*χ̅*±*s*), the counting data was expressed as (*n* (%)), and the comparison between groups was verified by two independent-sample t-test and *χ^2^* test, respectively. *P*<0.05 meant that the difference was statistically significant between groups.

## RESULTS

After 14 days of medicinal treatment, a comparison of the total effectiveness of treatment between groups suggested that it was higher in the study group than that of the control group, and the difference was statistically significant (*P*<0.05; Table-I). As shown in Table-II, after 14 days of medicinal treatment, the disappearance time of various ocular symptoms was shorter in the study group than that of the control group, and the difference was statistically significant (*P*<0.05).

**Table-I:** Comparison of the total effective rate of treatment between the two groups [*n* (%)].

Groups	*n*	Cure	Significant effective	Effective	Ineffective	Total effective rate
Control group	32	7 (21.88)	10 (31.25)	6 (18.75)	9 (28.13)	23 (71.88)
Study group	32	10 31.25)	15 (46.88)	5 (15.63)	2 (6.25)	30 (93.75)
*χ* ^2^	-	-	-	-	-	5.379
p	-	-	-	-	-	0.020

**Table-II:** Comparison of the time of disappearance of ocular symptoms between the two groups (*χ̅*±*S*, d).

Groups	n	Disappearance of corneal ulcer	Disappearance of photophobia and tearing	Disappearance of hypopyon	Disappearance of intra-ocular foreign body sensation
Control group	32	20.32 ± 3.54	21.55 ± 4.26	18.23 ± 3.11	16.46 ± 2.25
Study group	32	17.78 ± 3.39	17.97 ± 3.88	15.86 ± 2.23	14.71 ± 2.07
t		2.931	3.515	3.503	3.238
p		0.005	0.001	0.001	0.002

There was no significant difference in the comparison of the visual acuity level, keratitis severity score, corneal ulcer area and (1,3)-β-D-glucan level between the two groups before treatment (*P*>0.05). While 14 days after medicinal treatment, patients in the study group showed increased visual acuity level, narrowed corneal ulcer area, as well as decreased keratitis severity score and (1,3)-β-D-glucan level than those in the control group, with a statistically significant difference (*P*<0.05), as shown in Table-III. As described in Table-IV, there was no significant difference in the incidence of adverse reactions between the control group and the study group after 14 days of medicinal treatment (*P*>0.05).

**Table-III:** Comparison of ocular recovery before and after treatment between the two groups (*χ̅*±*S*).

Time	Groups	Visual acuity level	Keratitis severity score (points)	Corneal ulcer area (mm^2^)	(1,3)-β-D-glucan (ng/L)
Before treatment	Control group (*n*=32)	0.34 ± 0.11	5.32 ± 1.19	28.15 ± 7.45	6.57 ± 0.46
	Study group (*n*=32)	0.37 ± 0.15	5.14 ± 1.33	27.96 ± 7.67	6.41 ± 0.38
	*t*	0.912	0.571	0.101	1.517
	p	0.365	0.570	0.920	0.134
After treatment	Control group (*n*=32)	0.42 ± 0.18	1.14 ± 0.25	10.31 ± 2.44	3.78 ± 0.22
	Study group (*n*=32)	0.53 ± 0.24	0.97 ± 0.21	8.67 ± 1.85	3.22 ± 0.36
	t	2.074	2.945	3.030	7.508
	p	0.042	0.005	0.004	0.000

**Table-IV:** Comparison of the incidence rate of adverse reactions between the two groups [*n* (%)].

Groups	*n*	Irritating pain	Foreign body sensation	Photophobia	Endophthalmitis	Conjunctival congestion	Total incidence
Control group	32	1 (3.13)	1 (3.13)	2 (6.25)	0 (0.00)	1 (3.13)	5 (15.63)
Study group	32	0 (0.00)	2 (6.25)	1 (3.13)	0 (0.00)	1 (3.13)	4 (12.50)
*χ* ^2^	-	-	-	-	-	-	0.129
p	-	-	-	-	-	-	0.719

## DISCUSSION

FK is an ocular disease with a high rate of blindness and complicated pathogenesis, commonly including a history of ocular trauma with vegetative matter, unreasonable use of antibiotics or hormones, etc.^16^ FK has a slow progression and is difficult to diagnose. As the disease progresses to an advanced stage continuously, it may cause serious complications such as corneal perforation and endophthalmitis, which will seriously affect the patient’s eyeball structure and visual acuity, and even lead to blindness.^17^ Consequently, it highlights the crucial importance of timely diagnosis and effective treatment of this type of disease.

Currently, topical medication is a common therapeutic choice for FK, among which natamycin is a frequently used antifungal agent in clinic.^18^ In terms of the mechanism of action in the treatment of FK, the use of natamycin can promote the production of antibiotics-sterols by combining them with sterols in the fungal membrane. Consequently, it can damage thse fungal cytoplasmic membrane, enhance the permeability of the cell membrane, and further induce bacterial damage and apoptosis, leading to the killing of fungi eventually.^19^ However, combined treatment has been recommended in clinical practice since natamycin has a poor effect on some patients with severe fungal infections.^20^

In our study, on the basis of natamycin treatment, voriconazole was added to treat patients with FK. According to the results of our study, the combined use of natamycin and voriconazole in the treatment of FK patients can effectively remove ocular fungi, promote the improvement of ocular symptoms and visual acuity, and has good safety without influence in increasing the adverse reactions. Concerning the possible reason, voriconazole is a novel antifungal agent, and its anti-fungal activity is 50~100 times that of fluconazole. It can inhibit the demethylation of 14α-sterols induced by cytochrome P450 in fungi, and prevent ergosterol biosynthesis, so as to play a strong anti-fungal and fungi-killing effect.^21^

In addition, the self-made voriconazole eye drops applied in our study have high bioavailability, which can increase the drug concentration in the cornea, improve the anti-fungal activity and penetration to the cornea, inhibit and kill various intra-ocular fungi, so as to reduce the degree of corneal infection and improve ocular symptoms consequently.^22^ For instance, Li C et al.^23^ have reported that the novel voriconazole injection into the corneal stroma can significantly improve the visual acuity of patients with FK and reduce the risk of corneal perforation. Meanwhile, multiple domestic studies^24^-^26^ have also documented that voriconazole has a good effect against fungal infection, and it is widely used in the treatment of pulmonary fungal infection, invasive fungal diseases, etc.

Voriconazole can be regarded as an effective adjuvant in the treatment of FK. Simultaneously, natamycin itself is highly sensitive to Fusarium, Aspergillus and other fungi. Following intra-ocular application, this medical agent can form micropores on the cell membrane to increase the permeability of the cell membrane, promote the apoptosis of fungal cell components, and achieve anti-fungal and fungi-killing effects.^27^ Significantly, the combined use of natamycin and voriconazole can significantly enhance the anti-fungal effect, improve the sensitivity of the drug to fungi, effectively remove various intra-ocular fungi, control the condition of illness, and eventually effectively improve the corneal ulcer of patients.^28^,^29^ Besides, safety analysis of the combined treatment revealed that there was no difference in the incidence of adverse reactions between the study group and the control group (*P*>0.05).

### Limitations:

It suggests that the combination of natamycin and voriconazole may not increase the risk of adverse reactions to medication. It may be explained by the small sample size of this study. Therefore, in order to further clarify the safety of natamycin combined with voriconazole in the treatment of patients with FK, studies with a larger sample size and prolonged duration of follow-up are recommended to observe the recurrence of subsequent infection, so as to provide a more favorable reference basis for clinical practice.

## CONCLUSIONS

The combined use of natamycin and voriconazole for treating FK patients can effectively alleviate the ocular symptoms, completely eliminate ocular fungi, promote the recovery of the cornea and visual acuity, and improve clinical therapeutic effect eventually.

### Authors’ Contributions:

**JC and CY** designed this study prepared this manuscript, are responsible and accountable for the accuracy and integrity of the work.

**QW** **and HL** collected and analyzed clinical data.

**LA and RZ** Data analysis**,** significantly revised this manuscript.
